# Effect of Processing Parameters on Bonding Performance of a Carbon Fiber/Polyetheretherketone Thermoplastic Composite Prepared by Induction Welding

**DOI:** 10.3390/ma16113954

**Published:** 2023-05-25

**Authors:** Bo-Kyung Choi, Chang-Soo Kang, Myeong-Han Yoo, Min-Kang Seo

**Affiliations:** 1 Korea Carbon Industry Promotion Agency, Jeonju 54853, Republic of Korea; 2 Korea Institute of Convergence Textile, Iksan 54588, Republic of Korea

**Keywords:** thermoplastic composites, induction welding, polyetheretherketone

## Abstract

Among the various welding techniques used to bond thermoplastic composites, induction welding stands out as a fast, clean, and contact-free process that shortens the welding time and prevents the weight increase of mechanical fastening, such as rivets and bolts. In this study, we manufactured polyetheretherketone (PEEK)-resin-based thermoplastic carbon fiber (CF) composite materials at different automated fiber placement laser powers (3569, 4576, and 5034 W) and investigated their bonding and mechanical characteristics after induction welding. The quality of the composite was evaluating using various techniques, including optical microscopy, C-scanning, and mechanical strength measurements, and a thermal imaging camera was used to monitor the surface temperature of the specimen during its processing. The results revealed that the preparation conditions of the polymer/carbon fiber composites, such as the laser power and surface temperature, significantly affect the quality and performance of the induction-welding-bonded composites. A lower laser power during preparation resulted in weaker bonding between components of the composite and yielded samples with a lower shear stress.

## 1. Introduction

Thermoplastic composites are effective alternatives to thermosetting matrix composites and can be used in various applications, because they can be easily processed without cutting and have a long lifespan, low storage cost, and high recyclability, as well as good impact resistance, heat resistance, and flame retardancy [1,2,3,4,5]. In recent years, composite materials for aircraft structures [6,7] have been predominantly manufactured using polypropylene and polyamide. More recently, thermoplastic composite materials fabricated through automated fiber placement (AFP) and thermoforming techniques [8,9,10] using polyetheretherketone (PEEK) [11,12,13], polyetherketoneketone [14], polyaryletherketone [15], and polyphenylene sulfide [16] have attracted attention. Among thermoplastic resins, PEEK is widely used because of its high heat resistance and favorable mechanical properties, which are comparable to those of thermosetting epoxy [17,18]. Gao et al. [19,20] extensively studied the effects of cooling rate on the crystallinity, and interfacial, in-plane, and interlaminar properties of carbon fiber (CF)/PEEK composites. Further, studies have demonstrated that the fiber–matrix interphase adhesion and the bulk mechanical properties of thermoplastic composites can be optimized, if not completely maximized, by controlling their crystallinity, which is governed by processing conditions, such as the cooling rate. Further, Comer et al. [21] performed a comparative evaluation of CF/PEEK laminates processed using in situ laser-assisted automated tape placement and autoclave consolidation processes.

Owing to their advantageous features, PEEK composites can be used as components in the skin layers that are responsible for the aircraft shape. However, as the skin layer of an aircraft is an exposed part, it should be structurally rigid and have the ability to withstand the fatigue load generated by the repeated opening and closing of a hinge part. Therefore, such reinforcing panels should be bonded well to ensure their structural rigidity and strength.

Bonding is an important step in the manufacture of thermoplastic composites; however, the irregularities introduced into the structure during bonding can weaken the material. Although mechanical fastening and adhesive bonding methods can be used for metals and thermosets, they are not suitable for thermoplastics. Mechanical fastening leads to many disadvantages, including stress concentration in the material, peeling of the material during drilling, thermal expansion difference-induced stress between a fastener and composite, penetration of water into the joint, galvanic corrosion, weight gain, extensive labor, and high time requirements. Adhesive bonding is superior to mechanical fastening because it avoids stress concentration; however, its application is complicated because adhesives are difficult to bond with chemically inactive thermoplastic resins [22,23,24,25].

Owing to the abovementioned issues, fusion welding techniques (such as ultrasonic welding (UW), resistance welding (RW), and induction welding) are commonly used to bond thermoplastic composites. UW allows fast cycle times of less than 1 s and does not require the use of an external non-polymer-based material, such as a susceptor. However, the joint thickness is limited, and the process is sensitive to surface conditions and temperature [26,27]. RW, which welds the entire welding area at once, has been widely used because of its excellent performance in the processing of highly conductive materials or thin plates. However, RW has certain drawbacks, such as significant non-uniform temperature distribution at the weld interface, high force to be applied to the adherends, difficulty in maintaining a uniform pressure at the weld area, and a high power requirement [28]. Induction welding has attracted considerable interest because it can overcome the limitations associated with adhesive bonding while preventing increases in the welding time and weight of the welded products. Lionetto et al. [29] used several numerical simulations to select a processing window as a function of coil speed and current for the welding of CF/PEEK joints, and Wang et al. [30] designed and investigated the temperature field of the bonding area using different current parameters.

**Figure 1:**
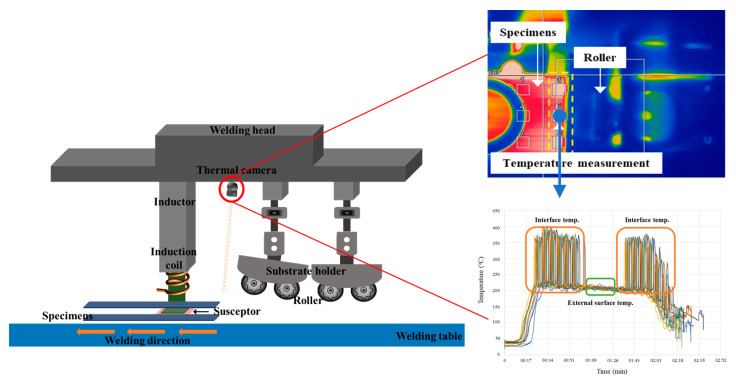
Schematic of the induction welding system.

Induction welding is a noncontact, fast, and clean process that provides a less contaminated surface, thereby enabling continuous welding and constant-temperature control, which are required for automated processing [31,32,33,34,35,36]. Various materials, such as the CF prepreg and fabric, metallic meshes, and ferromagnetic particles, have been used as heating elements for the induction welding of thermoplastic composites. Stainless steel is commonly used as a susceptor in thermoplastic composite induction welding. The induction coil and susceptor are connected to the inductor, so the welding continues in the welding direction. Therefore, the susceptor does not remain at the interface but moves along with the welding direction. The technique is particularly applicable to complex shapes, and it facilitates both localized heating and heating process control via a susceptor. During the induction welding process, an eddy current generated by the magnetic field from an induction coil heats the susceptor, which melts the two components. The molten components blend and are welded when solidified under pressure. Figure 1 shows a schematic of the induction welding system.

In this study, to assemble high-performance thermoplastic composites for aviation, thermoplastic composites were fabricated using different AFP laser powers (3569, 4576, and 5034 W), and an induction welding process was performed. The induction-welded thermoplastic composites were analyzed through optical microscopy, C-scanning, and single-lap shear strength testing. The surface temperatures of various samples were monitored using a thermal imaging camera.

## 2. Experimental

### 2.1. Materials and Sample Preparation

Unidirectional (UD) CF/PEEK tape used for manufacturing the thermoplastic composites was supplied by Toray (CETEX^®^ TC1200 PEEK); it had a fiber areal weight of 145 g/m^2^ and width of 305 mm (AS4D fiber with a volume fraction of 59%). PEEK is a semi-crystalline polymer with a typical crystallinity degree of 34% and glass transition (*T*_g_) and melting (*T*_m_) temperatures of approximately 143 and 343 °C, respectively. Table 1 lists the mechanical properties of CF/PEEK composites provided by the manufacturer.

**Figure 2:**
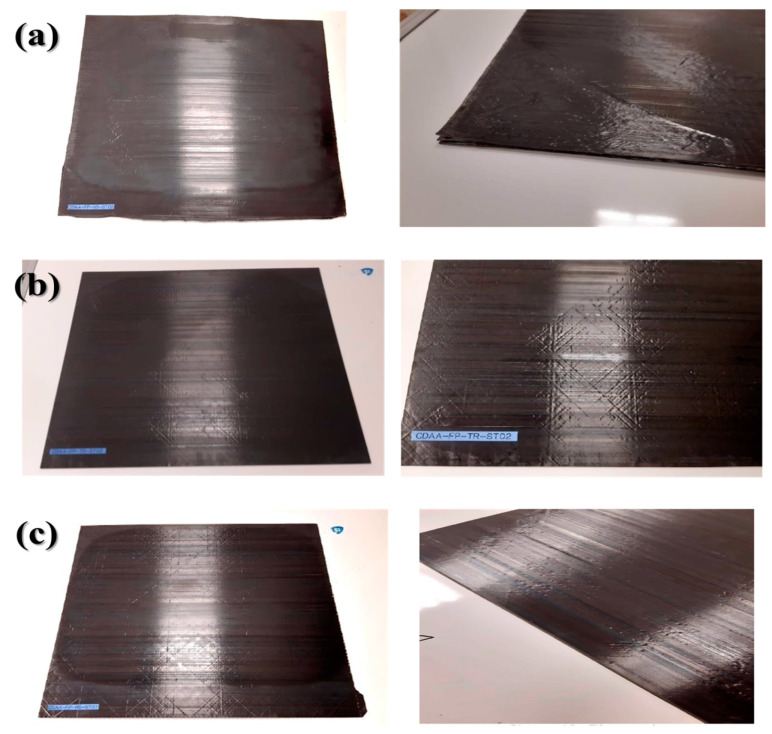
Digital photographs of flat-panel specimens processed using different laser powers: (**a**) 3569 W, (**b**) 4576 W and (**c**) 5034 W.

Figure 2 shows the digital photographs of the flat-panel specimens manufactured using an AFP machine (Coriolis Co., Queven, France). The selected stacking sequence for all laminates was (0/−45/0/45/90/45/0/−45)_16_ and the dimensions and thickness were 700 mm × 600 mm and 2 mm, respectively. Three different preparation conditions were chosen for fabrication, with varying AFP laser power and surface processing temperatures, as listed in Table 2. Lim et al. [38] studied the application of laser surface treatment on the CF reinforced thermoplastic polymer surfaces to enhance adhesive bonding strength and investigated the underlying mechanism. When using a high-power laser treatment (>22.5 W), the adhesive layer was unable to fully impregnate the exposed fibers, resulting in a failure to form a proper interface with the composite matrix. Thermal damage, such as delamination, matrix cracking, and fiber/matrix debonding, was also observed on the composite adherend due to high-power laser. This is because the heat absorbed by the CF is conducted to the surrounding area [39]. Therefore, as the laser power increases over 5100 W, it is expected that the performance will degrade due to the deterioration of the matrix.

### 2.2. Hot-Pressing Process

The flat-panel specimens were molded into CF/PEEK composites using a hot-pressing process involving a heating and pressurization cycle to melt the resin, followed by a cooling cycle for consolidation. The heat treatment of the flat-panel specimens was performed by hot pressing at 380 °C for 30 min at a pressure of 9–50 bar, and cooling at 5 °C/min to room temperature. CF/PEEK composites with an average density of 1.55 g/cm^3^ were obtained. In this manuscript, we will compare the morphology and mechanical behavior of two cases (Only AFP vs. AFP + hot press).

### 2.3. Induction Welding

The induction welding setup consisted of an induction heating system, a roller for applying pressure, a welding jig, and a thermal imaging camera. The heating system included a pancake-type induction coil and power supply device. Power was supplied (Ambrell Easy Heatmachine, Minneapolis, MN, USA) by a 10 kW device at a frequency in the range of 150 to 450 kHz and the maximum coil and susceptor, and Joule-based fiber heating occurred within the prepreg fibers. The process temperature, measured with a pyrometer located directly on the susceptor, was maintained at 570–600 °C; the average temperature of the sample surface was 240 °C, the speed of the robot was 5.4 mm/s, and the pressure was maintained at 2 bar. By maintaining consistent air flow and continuous temperature control using the thermal imaging camera, the temperature in the welding region could be increased above the melting temperature of the PEEK matrix, i.e., up to 400 °C, whereas the temperature of the upper surface of the joint was maintained below 250 °C.

**Figure 3:**
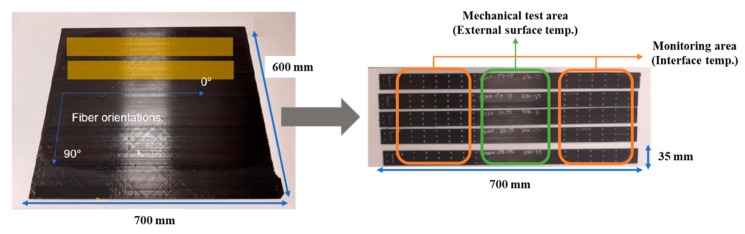
Digital photographs of the five samples extracted from one panel.

Figure 3 shows digital photographs of the five samples (700 mm × 35 mm) extracted from one panel for induction welding. The samples were dried at 95 and 115 °C for 4 h in an oven before each welding. Owing to in situ monitoring of samples (through holes), it was possible to obtain temperature curves with an approximation of the temperature at the interface (throughout the holes) and on the external surface (between the holes) using the thermal imaging camera.

### 2.4. Characterization

An optical digital image analysis system equipped with a digital camera (KCX-20B, Korea Lab Tech., Shanghai, China) and a scanning electron microscope (SEM; AIS 2000 C, Seron Tech. Inc., Uiwang, Korea) were used to observe the morphologies of the thermoplastic composites. Further, the thermoplastic composites were subjected to ultrasonic C- and B-scanning (SDI 5380, CBOL Corporation, Los Angeles, CA, USA) to detect the presence of defects (pores or voids). Microfocus X-ray computed tomography (SMX-225CTS, Shimadzu, Kyoto, Japan) was conducted for the nondestructive visualization of the composite interiors to estimate their void content. The micro-CT instrument was equipped with a flat laminate detector (512 × 512 pixels) and a microfocus X-ray source (170 kV and 190 μA). The obtained void ratios were compared with those determined through acid digestion. Acid digestion of the CF/PEEK composites was performed according to the ASTM D 3171 standard [40]. This method of void content assessment involves the degradation of the matrix with a solution of sulfuric acid (H_2_SO_4_) to extract the CFs and determine the mass of the dry fibers. The void ratio obtained by acid digestion was determined using a minimum of three specimens from each composite part. The void ratio was calculated as follows:

(1) $$V_{p}\left(\%\right)=1-\left(V_{f}+V_{r}\right)$$

where $V_{f}=\frac{m_{f}\times \rho_{s}}{m_{s}\times \rho_{f}}\times 100$, $V_{r}=\left(1-\frac{m_{f}}{m_{s}}\times 100\right)\times \frac{\rho_{s}}{\rho_{r}\;},\;$*m_f_* is the mass of the fibers, *m_s_* is the mass of the specimen, $\rho_{s}$ is the specimen density, $\rho_{r}$ is the matrix density (1.30 g/cm^3^), and $\rho_{f}$ is the fiber density (1.80 g/cm^3^). Moreover, $\rho_{s}$ was measured based on Archimedes’ principle using distilled water following ASTM D 792 [41], which includes the inner void structure.

**Figure 4:**
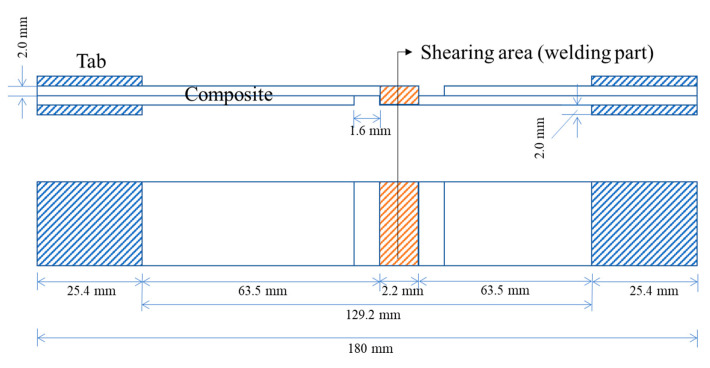
Schematic of the single-lap shear test sample.

Tensile tests were conducted to evaluate the mechanical properties of the composites. Samples were cut from the laminate and tabbed with the same composite laminate coupons using high-strength epoxy glue film (FM 73 adhesive, Solvay, Brussels, Belgium). All tests were performed using a universal testing machine (Instron 5982, Norwood, MA, USA) equipped with a 100 kN load cell. The tensile tests of at least five samples of each composite were conducted according to the ASTM D 3039 standard at a crosshead speed of 2 mm/min. Moreover, a single-lap shear test was performed after induction welding according to the Airbus AITM 1-0019 test method [42]. Figure 4 presents a schematic of the sample used for the single-lap shear test.

## 3. Results and Discussion

### 3.1. Characteristics of Composite Panels before Induction Welding

**Figure 5:**
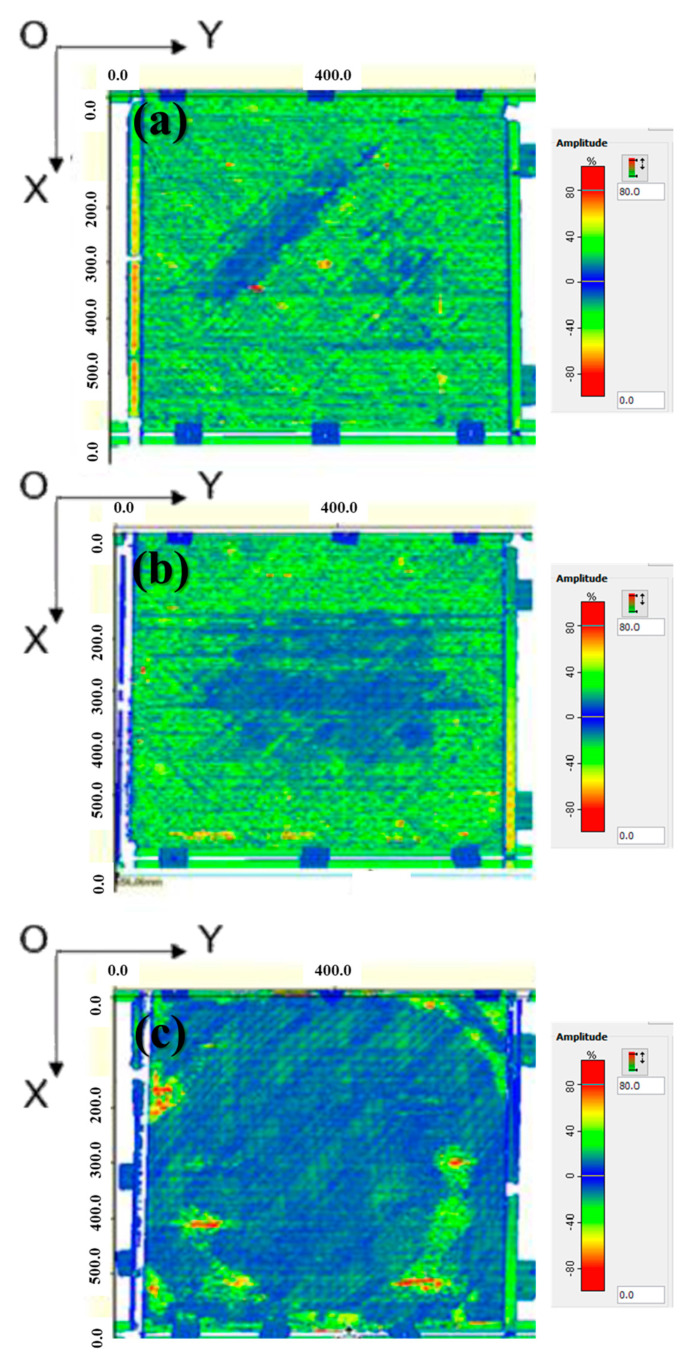
C-scan images of the CF/PEEK composites processed at (**a**) 3569 W, (**b**) 4576 W, and (**c**) 5034 W.

Figure 5 shows the C-scan images of the CF/PEEK composites. In this study, the distribution of the defects present in the interlayer separation region was analyzed according to the panel depth; this involved an assessment based on the color difference between the C-scan images. In the composite panel, adhesion was observed throughout the scan area (blue and green areas); however, a slight adhesive separation was observed in the red and yellow areas of the edge part but not at the center of the panel or the entire scan area. Compared with those of Figure 5a,b the adhesion of the interfacial layer in Figure 5c was clearly enhanced. This finding is consistent with the visual inspection results of the CF/PEEK composites. It proves that there is little difference between the center and edge of a panel.

Ma et al. [43] provided a mechanism of the fiber–matrix interaction in CF/PEEK composites. The micro-morphology of the fiber surface of the specimen fabricated at 405 °C under 0.5 MPa had a higher molding temperature than the specimen fabricated at 360 °C under 0.25 MPa and allowed the PEEK matrix to diffuse and strongly adsorb to the fiber surface. The flattened crystalline lamellar chains with high crystallinity in the interfacial region enable more polar components to interact functionally with the fiber surface. In contrast, a lower molding temperature reduces the viscosity of the molten PEEK matrix. Due to the crosslinking of PEEK, the mobility of crystallizable unit molecules is further reduced, which affects the nucleation mechanism and slows down crystal growth. Thus, it has been reported that the PEEK matrix is unable to form strong bonds with the fiber surface, as in the case of the specimen fabricated at 360 °C and 0.25 MPa.

The void contents of the composites are listed in Table 3. The average void content measured by X-ray tomography was 0.27–22.14%, whereas that measured by the acid digestion method was lower at 0.24%. Di Landro et al. [44] reported differences in void content when calculated using acid digestion compared to micro-CT imaging. Acid digestion is a well-established technique widely used in material characterization, but it can be time-consuming and costly. Micro-CT imaging is limited to capturing a cross-sectional view of each coupon, which can introduce inherent biased errors. However, microscopy has the advantage of providing information about the size, location, and shape of voids or other defects. The highest-quality CF/PEEK composites were achieved at an AFP laser power of approximately 5000 W. The variation in the quality of the samples is attributed to the difference in the AFP laser power and surface temperature during the preforming stage.

**Figure 6:**
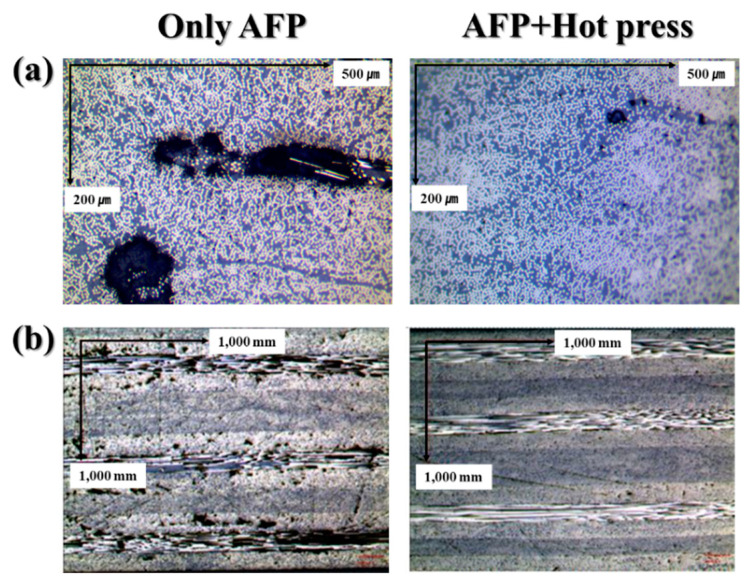
Cross-sectional (**a**) and plane-view (**b**) SEM images of the CF/PEEK composites processed at 5034 W.

Cross-sectional and plane-view SEM images of CF/PEEK composites subjected to only AFP presented in Figure 6 have considerably greater black areas compared with the sample subjected to AFP followed by hot pressing. These black areas correspond to porosities and suggest that the CF/PEEK composites processed by only AFP were not well consolidated compared to those subjected to AFP and hot-pressing processes.

**Figure 7:**
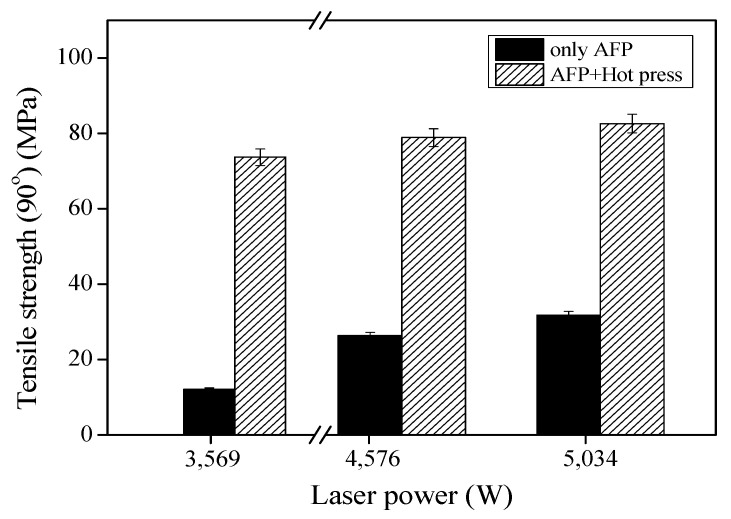
Tensile strength (90°) of the CF/PEEK composites before and after hot pressing.

Figure 7 presents the 90° tensile strength of the CF/PEEK composites with respect to the AFP laser power before and after hot pressing. Upon processing at 5034 W laser power, the tensile strength of the preform specimen manufactured with only AFP was as low as 31.8 MPa, and it increased to 82.6 MPa after hot pressing. The tensile strength (90°) specimen was approximately 82.6 MPa, which is significantly lower than the tensile strength (0°) specimen, accounting for only about 3.4% (2400 MPa). These results indicate that, in the case of the tensile test (90°) specimen, the CF imparting the reinforcing effect is positioned perpendicularly or laterally to the direction in which the tensile load is applied. The resistance to the tensile load applied to the composite specimen is mainly due to the relatively weak matrix. In addition, as the laser power increased, the tensile strength of the preform specimen increased both before and after hot pressing. Because of the rapid warming and cooling of the specimens during the AFP process and the high viscosity of the PEEK substrate, defects such as poor interfacial bonding between the fibers and substrate, incomplete crystallization, and internal pores were observed in the AFP-processed specimens. Hot pressing enables the polymer to have a longer diffusion time to complete its nucleation and growth processes. During compression, PEEK recrystallization could occur, enabling additional bonding within the adhesive (cohesive) between the fiber matrix and PEEK fibers. Overall, the tensile strength of the composite was confirmed to improve after hot pressing. Saenz-Castillo et al. [45] studied three different out-of-autoclave processes, including vacuum bag-only, hot pressing, and in situ consolidation (ISC), to compare the mechanical response and porosity of samples processed by various manufacturing methods. The results of differential scanning calorimetry revealed the degree of crystallinity of the manufactured laminates [45]. The laminates obtained by the ISC method presented a slightly lower degree of crystallinity (36.9%) than those manufactured by hot pressing (crystallinity degree: >42.7%). The final degree of crystallinity of thermoplastic composites is mainly related to the cooling rate of the manufacturing process. Therefore, high cooling rates during ISC are the main cause of the lower degrees of crystallinity of the ISC-manufactured laminates.

### 3.2. Characteristics of Composite Panels after Induction Welding

**Figure 8:**
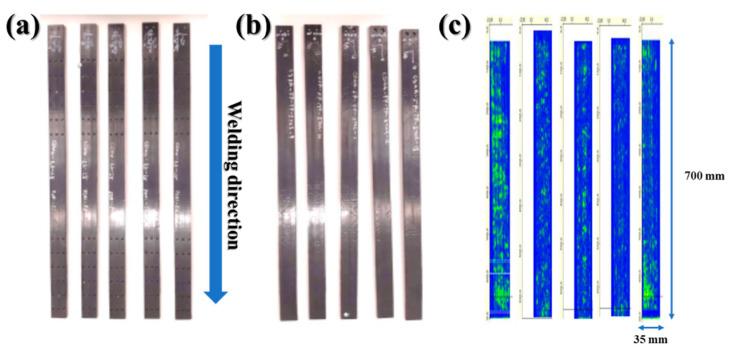
Digital photographs of specimens after whole-area welding: (**a**) external surface–top, (**b**) external surface–bottom, and (**c**) C-scan images.

The thicknesses of the CF/PEEK composites manufactured at 5034 W laser power before and after whole-area welding are listed in Table 4. As shown, the thicknesses of all samples before and after induction welding differed by approximately ±0.2 mm, which is comparable to the theoretical values. As illustrated in Figure 8, no major defects were observed in the upper, lower, and outer surfaces of the specimens owing to induction welding.

**Figure 9:**
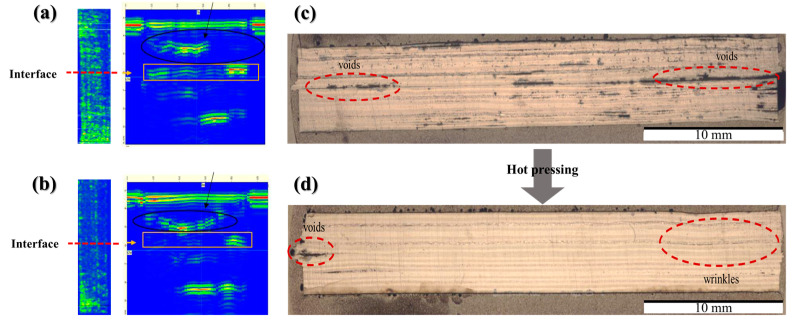
B-Scan (**a**) and (**b**) and optical micrographs (**c**) and (**d**), respectively.

Figure 9 shows the B-Scan (a and b) and optical micrographs (c and d) of the section interfaces of the composite welded specimens manufactured at 5034 W laser power. In Figure 9a, several reflective images (yellow or red areas) are observed at the center of the welding surface of specimens; this can be attributed to the incidence and repeated reflectance of both reflective and other waves in the voids within the composite. In Figure 9b, no or minor defects were revealed in the welded composite panel, confirming the success of the welding. The dark, irregular shapes representing voids are observed slightly on the left and right sides, while the interlayer interfacial bonding appears to be relatively well-established at the center of the specimen.

**Figure 10:**
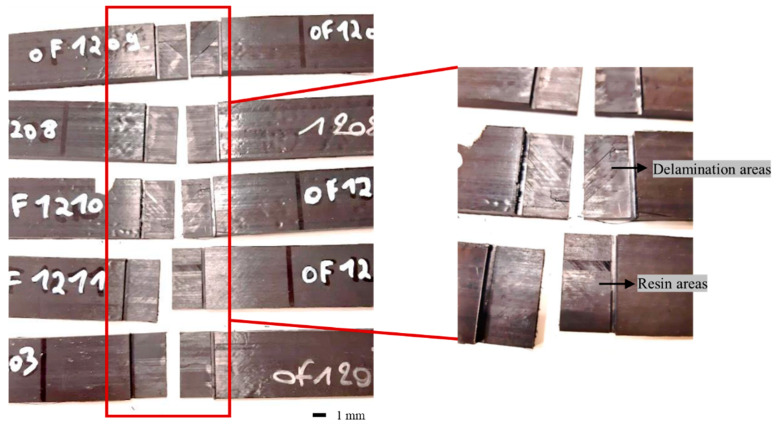
Digital photographs after the single-lap shear strength test of the manufactured composite welded specimens.

Table 5 lists the results of the single-lap shear test conducted to evaluate the bonding characteristics of the composite specimens after part-area welding specimens manufactured at 5034 W laser power. The observation of the fracture surface after the test reveals that the composite materials underwent “cohesive failure” and were broken as a result. The results of the nondestructive and single-lap shear tests presented a similar trend. The average single-lap shear strength of the welded specimen is 24.68 MPa, with a standard deviation of 0.5 MPa. Reis et al. [46] reviewed the effectiveness of induction welding in several thermoplastic composites. They evaluated the lap shear strength values, which ranged between 14 MPa and 43 MPa. Lionetto et al. [30] obtained similar test results for the induction welding of CF/PEEK composites. Moreover, it is considered that the voids act as defects, regardless of their position, and affect the physical properties of the material, thereby causing a decrease in the shear strength of the composite. The presence of defects induced at the edges of the interface can be attributed to the degradation of the polymer above the high induction-welding molding temperature. Figure 10 shows digital photographs of the composite welded specimens after the single-lap shear strength test. The failure of the specimen leads to notable resin areas at the tip of the weld and distinct delamination propagation (light-colored zone). Zhang et al. [47] reported that hot pressing enables the polymer to have a longer diffusion time to complete nucleation and crystal growth. Hot pressing facilitates better matrix infiltration into the fibers, and ultimately, a stronger CF and PEEK bonding interface stabilizes the crystal structure and fiber reinforcement during the shear stress process of the specimens. Consequently, a greater shear strength is achieved.

## 4. Conclusions

In this study, thermoplastic composites were fabricated using CF/PEEK prepreg at different laser powers, and the bonding characteristics of the composites were investigated after each induction welding.

(1) The highest quality CF/PEEK composites were achieved at the AFP laser power of 5034 W. The void content estimated by X-ray tomography was 0.27%, whereas that measured by the acid digestion method was not detected.
(2) The 90° tensile strength of the specimen manufactured with hot pressing was as low as 31.8 MPa, and it increased to 82.6 MPa after hot pressing.
(3) The thicknesses of all the samples were found to be similar to the theoretical values, with no significant difference observed (±0.2 mm) between the thicknesses measured before and after induction welding.
(4) The C- and B-scanning results confirmed that the specimen before hot pressing had more defects than the specimen after hot pressing, in agreement with the results of the tensile strength test. Moreover, cohesive failure led to the fracture of the composites under load, and the average shear strength was 24.68 MPa.
(5) The average single-lap shear strength of the welded specimen was 24.68 MPa, with a standard deviation of 0.5 MPa. The observation of the fracture surface after the test revealed that the composite materials underwent “cohesive failure” and broke.

The results of this study clearly indicate that, when bonding thermoplastic composite materials by induction welding, the thermal characteristics and contact between the samples have a more significant effect on their bonding and mechanical characteristics. In the future, we plan to further optimize the conditions of induction welding for achieving better bonding of the components. Additionally, the proposed induction welding method for bonding thermoplastic composites can be upscaled to verify its practical mass production capability.

## Figures and Tables

**Table 1:** Mechanical properties of the CF/PEEK composites provided by the manufacturer [37].

Property	Test Method	Strength Value (MPa)
Tensile strength (0°)	ASTM D 3039	2410
Tensile strength (90°)	ASTM D 3039	86
Compressive strength (0°)	ASTM D 6641	1300

**Table 2:** Parameters of the AFP conditions.

Sample	Laser Power (W)	Surface Temperature (°C)	Compaction Force (N)	Robot Speed (m/s)
(a)	3569	275	1000	0.2
(b)	4576	325		
(c)	5034	385		

**Table 3:** Void ratios of the CF/PEEK composites determined through different methods.

Sample	X-ray Tomography (%)
(a)	22.14
(b)	4.46
(c)	0.27

**Table 4:** Thicknesses of CF/PEEK composites manufactured at 5034 W laser power before and after whole-area welding.

Welding Specimen	Theoretical Thickness (Upper + Lower) (mm)	Real Thickness After Welding (mm)	Difference between Theoretical and Real Thickness (mm)
#1	4.66	4.88	0.23
#2	4.65	4.85	0.20
#3	4.67	4.77	0.10
#4	4.83	4.83	0.00
#5	4.65	4.73	0.08

**Table 5:** Single-lap shear strength test results after part-area welding specimens manufactured at 5034 W laser power.

Welding Specimen	Avg. Single-Lap Shear (MPa)
#1	20.6
#2	24.6
#3	26.1
#4	28.0
#5	24.1

## Data Availability

Not applicable.
